# Profiles and Influencing Factors of Work–Family Balance Among Nurses in China: A Cross-Sectional Study Based on Latent Profile Analysis

**DOI:** 10.1155/jonm/8556545

**Published:** 2025-02-17

**Authors:** Qian Tang, Yijia Wang, Chi Zhang, Yuefan Zhao, Xinjie Zhou, Jie Huang, Jun Yao

**Affiliations:** ^1^School of Nursing, Nanjing Medical University, Nanjing, Jiangsu, China; ^2^School of Health Policy and Management, Nanjing Medical University, Nanjing, Jiangsu, China; ^3^Jiangsu Provincial Institute of Health, Nanjing Medical University, Nanjing, Jiangsu, China

**Keywords:** latent profile analysis, nurses, work–family balance, work–family conflict

## Abstract

**Aim:** To profile the work–family balance (WFB) among clinical nurses and identify their influencing factors.

**Background:** WFBs take on various patterns in nurses. Nurses with different characteristics exhibit various patterns of WFB.

**Methods:** A cross-sectional study was performed, involving 1292 nurses from public hospitals in Jiangsu Province from February to April 2024. Latent profile analysis (LPA) was employed to categorize the data, and multinominal logistic regression analysis to determine factors influencing WFB.

**Results:** A total of 1718 questionnaires were distributed, yielding 1292 valid responses (a response rate of 75.2%). The total score of WFB was 44.35 ± 8.693 points. The LPA revealed three profiles of WFB: the family priority group (28.1%), the balanced adaptation group (46.8%), and the challenge coping group (25.1%). The entropy value was high (0.892), indicating a correct classification. Multivariate regression analyses showed that professional title, department, nonclinical work pressure, organizational identification, work meaning, and self-efficacy as factors influencing WFB profiles.

**Conclusion:** The WFB among nurses was at a moderate level with significant heterogeneity and could be divided into three profiles. The challenge coping group presented lower professional titles and higher self-efficacy, and working at surgical, pediatric, and other specialized departments. Managers can tailor intervention strategies based on the characteristics and influencing factors of each profile.

**Implications for Nursing Management:** Nursing managers must consider the characteristics of high-risk groups and factors that affect WFB and take targeted measures to mitigate work–family conflicts (WFC) among nurses and stabilize the nursing workforce.

## 1. Introduction

In the context of ongoing development and transformation within the healthcare system, particularly since the breakout of COVID-19 pandemic, the nursing workforce faces unprecedented challenges in work and family [1], including huge workload, serious staff shortage, and no time to undertake personal and familial responsibilities [2]. In this context, maintaining work–family balance (WFB) is of great significance for enhancing their performances in hospitals and accomplishing their duties in families, which in turn increases the benefits of the healthcare system.

WFB indicates minimal conflict and maximal reciprocity between work and family [3]. A work–family relationship is manifested with work–family conflict (WFC) and family–work conflict (FWC), but also work–family facilitation (WFF) and family–work facilitation (FWF) [4]. Prior studies have emphasized the conflicts between work and family but overlooked their mutual facilitation [5, 6]. Therefore, it is necessary to comprehensively understand this complex relationship and explore factors that can enable WFB.

The previous research shows that half of the nurses face long-term conflicts between work and family, while 11% experience a persistent impact of family on their work [7]. WFB of nurses is not unidimensional but may manifest multiple types [8–10]. The spillover theory [11] reveals that the intricate interconnectedness between work and family, where emotions, attitudes, skills, and behaviors formed at work can both influence and be influenced by familial factors. This interconnection can be either positive, benefiting family life through work satisfaction and skills, or negative, inducing family conflict due to work-related exhaustion.

WFB is jointly influenced by work-related factors, family-related factors, and individual characteristics [4, 12, 13]. Work-related factors include department, workload, organizational identification, and work environment. The extensive literature indicates that medical personnel in departments with long-term high workload, such as emergency departments, ICUs, and operating rooms, are more prone to WFC [14, 15]. COVID-19 poses more challenges on nurses in work, such as adverse consequences of COVID-19, large number of patients, long-term separation from family members, and issues related to new policies and procedures [2]. These challenges increase the odds of WFC, impair their mental health [16, 17], reduce their job satisfaction [8, 18, 19], and enhance their intentions of changing jobs [20–22]. In order to enhance job satisfaction and staff retention, employers and managers need to recognize the impact that achieving WFB may have on future workforce sustainability. Studies showed that reducing work stress [23], enhancing organizational identification [24], and improving the work environment [25] can significantly decrease the risk of WFC and increase the chance of WFB.

Family-related factors, such as the number of children [9] and marital status [12], can contribute to individual WFC. From the perspective of individual characteristics, demographic variables such as age [26, 27], gender [28, 29], and education level [8], as well as personality, significantly influence the level of WFC. A higher age and a senior professional tile may lead to WFC. The research has also demonstrated a positive correlation between WFB and employees' self-efficacy [30]. Variable-centered analyses (e.g., regression analysis and confirmatory factor analysis) have been conducted to explore the outcomes and influencing factors of WFC among nurses [28]. However, these studies often focus on one dimension of WFB.

Latent profile analysis (LPA) [31], an individual-centered statistical method, can be used to examine the heterogeneity in individuals' responses to classify subpopulations with similar characteristics or behaviors [9, 10]. Furthermore, it can explore the influencing factors associated with different categories. LPA has been applied in various fields, including psychology, education, sociology, and healthcare. In recent years, latent profile models have been applied to work–family interface research. For instance, one study has identified three types of WFC among married nurses: low-conflict type, work-dominant conflict type, and high-conflict type [9].

Using LPA, this cross-sectional study aims to uncover the characteristics and influencing factors of WFB among nurses in China. The goal is to provide a reference for employers and managers to develop targeted support strategies for nurses' WFB.

## 2. Subjects and Methods

### 2.1. Study Design and Participants

A cross-sectional study was conducted among nurses from public hospitals in Jiangsu, China, from February to April 2024. Convenience sampling was adopted. Nurses holding valid nursing licenses, actively engaged in clinical practice, and informedly volunteering for participation were included. Nurses who were on vacation or ready to undergo further training during the study were excluded. Using G∗ Power V.3.1.9.7, a minimum sample size of 336 was calculated based on the multiple regression analysis (effect size of 0.10, power of 0.95, significance level of 0.05, and predictors of 22). Finally, 1292 valid questionnaires were successfully gathered, meeting the size required for the LPA (at least 500) [32].

### 2.2. Measurements

#### 2.2.1. Demographic Characteristics

Selected demographic characteristics included age, gender, marital status, number of children, years of work experience, education, employment type, professional title, hospital level, department, personal annual income, commute time, and nonclinical work stress.

#### 2.2.2. WFB Scale (WFBS)

The Chinese version of the WFBS revised by Zeng and Yan [33] was used to measure WFB in its four dimensions: work-to-family conflict, family-to-work facilitation, family-to-work conflict, and work-to-family facilitation. A five-point Likert scale ranging from 1 (*strongly disagree*) to 5 (*strongly agree*) was used to score the 14 items in each dimension. In the dimensions of work-to-family conflict and family-to-work conflict, a higher score indicates that a more negative effect of work on family or family on work; in the dimensions of work-to-family facilitation and family-to-work facilitation, a higher score indicates that a more positive effect of work on family or family on work. In this study, Cronbach's alpha coefficient of the scale was 0.821.

#### 2.2.3. Organizational Identification Scale

The Organizational Identification Scale developed by Grzywacz and Marks [34] was used to evaluate nurses' sense of identity in their hospitals or departments. This scale comprises six items scored on a five-point Likert scale ranging from 0 (*completely inconsistent*) to 4 (*completely consistent*). A higher score indicates a stronger sense of being recognized by the hospital/department. Cronbach's alpha for the Organizational Identification Scale was 0.956 in the current study.

#### 2.2.4. Generalized Self-Efficacy Scale (GSES)

The Chinese version of the GSES was utilized to measure individuals' general beliefs in their effectiveness in coping with difficult situations [35]. It has 10 items and is a four-point Likert scale ranging from 1 to 4. The total scores are categorized as follows: *low self-efficacy* (10–20), *moderate self-efficacy* (21–30), or *high self-efficacy* (31–40). The higher the score, the stronger the nurse's self-efficacy in facing challenges. In this study, Cronbach's alpha for the GSES was 0.959.

#### 2.2.5. The Work and Meaning Inventory (WAMI)

The WAMI developed by Steger et al. [36] in 2012 was adopted to assess nurses' perception of the value and importance of their work. The WAMI comprises three dimensions. Positive meaning was evaluated by questions 1, 4, 5, and 8; meaning-making through work by questions 2, 7, and 9; greater good motivations by questions 3, 6, and 10. Each of these 10 items is scored on a Likert scale ranging from 1 to 5, in which the third question “*My work makes no difference to the world*” is scored in a reverse order. A higher score indicates a stronger sense of professional identity and work significance. In this study, Cronbach's alpha for this scale was 0.923.

### 2.3. Data Collection

The data were collected through an anonymous and self-reported questionnaire through the “Questionnaire Star” network platform (https://www.wjx.cn). The team leader liaised with nursing department directors across hospitals, explaining the purpose of the survey and the method of completing the questionnaire. Collaborating with hospital administrators, the QR code and online questionnaire link were disseminated via WeChat to clinical nurses. Consent and clear instructions were provided at the beginning of the questionnaires. To ensure the completeness of the questionnaire, each question was set as mandatory during creation, and each IP address could only be entered once to prevent duplication. Two uniformly trained investigators continuously monitored the recovery data. Once no new data were generated for consecutive 7 days, the data were exported. Then, two researchers double-checked the quality of the questionnaire and deleted those that violated the filling requirements or were completed within 240 s.

### 2.4. Statistical Analysis

In the first step, LPA was conducted using Mplus 8.3 software to examine the types of WFB. The scores from the 14 items of the WFBS were used as manifest variables. The number of latent profiles was increased from 1 to 5, and the fit of the final model was selected based on the following three criteria. (a) Information evaluation criteria: Model fit was assessed by comparing the disparities between expected and observed values through the Akaike information criterion (AIC), Bayesian information criterion (BIC), and adjusted BIC (aBIC). A smaller value indicates a better model fit [37]. (b) Classification evaluation criteria: The entropy value represents the accuracy of classification ranging between 0 and 1. A value above 0.80 indicates a classification accuracy exceeding 90%, and a value closer to 1 indicates a more precise classification [38]. (c) Likelihood ratio tests: The likelihood ratio test indices (Lo–Mendell–Rubin, LMR) and the bootstrap likelihood ratio test (BLRT) based on bootstrap were used to assess the fit differences between *k* − 1 and *k* profile models. A *p* value less than 0.05 indicates that a *k* model significantly outperforms a *k* − 1 model [32]. These criteria only provide a reference for deciding a profile, and the interpretability of each profile was considered in determining the optimal mode. Each model was also evaluated on interpretability and sample size, with profiles containing less than 5% of the sample considered spurious [39].

In the second step, after the optimal model was determined, the characteristics of the latent profile were summarized, according to the conditional probability of each explicit variable, to determine and name the latent type of WFB. The latent profile of one nurse was inferred by calculating the posterior probability.

In the third step, SPSS27.0 statistical software was used for data analysis. The latent WFB profile was used as the grouping variable. Quantitative data were presented as mean and standard deviation (SD), and comparisons between groups were made using one-way ANOVA. Categorical data were described as frequency and percentage (%), and comparisons between groups were performed using a chi-square test. The impacts of nurses' demographic characteristics, work–family characteristics, organizational identification, self-efficacy, and work meaning on WFB profile were analyzed and described the characteristics of each profile.

In the fourth step, the statistically significant variables in the third step were used as independent variables and latent profiles as dependent variables in a multinominal logistic regression analysis. The significance level was set at *α* = 0.05, with *p* < 0.05 indicating statistically significant difference.

### 2.5. Ethical Considerations

The studies involving human participants were reviewed and approved by the Ethics Committee of Nanjing Medical University ([2024] No. 752).

## 3. Results

### 3.1. Characteristics of Participants

A total of 1718 questionnaires were collected initially. After screening, 1292 valid questionnaires remained, with an effective response rate of 75.2%. Of them, 95.4% were female, 64.4% aged 44 years or younger, and 79.8% had a bachelor's degree. These demographics are presented in Table 1.

### 3.2. LPA of WFB

The LPA was conducted using scores from the WFBS, and five latent profile models were fitted. The indices for fitting each model are shown in Table 2. As the number of latent profiles increased from one to five, the values for AIC, BIC, and aBIC decreased. The *p* values for LMRT and BLRT were significant (*p* < 0.05). However, the sizes in 4-profile and 5-profile models represented less than 5%, rendering their results uninterpretable. Therefore, the 3-profile model was selected as the optimal model for the following reasons: (a) the monotonic decreasing trend in AIC, BIC, and aBIC; (b) the entropy value greater than 0.8, indicating that at least 90% of individuals were correctly classified; and (c) significant LMRT and BLRT values. In this 3-profile model, the average probability of assignment to the latent profile ranged from 94.4% to 96.7%, indicating the credibility of the three latent profiles.

**Figure 1:**
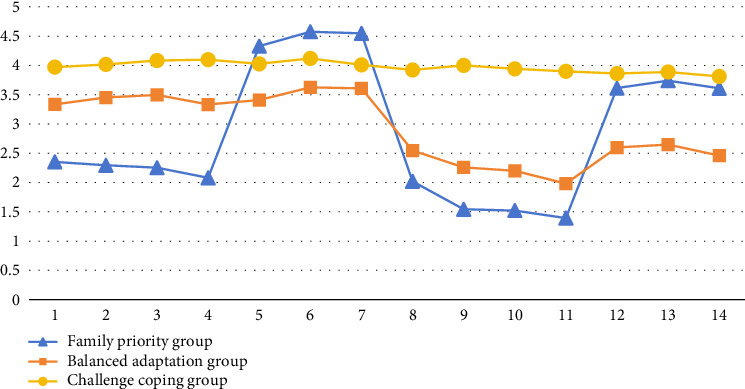
Characteristics of three WFB profiles.

Based on this 3-profile model, the scores of the 14 items of WFBS were obtained (Figure 1). Profile 1 comprised 363 cases (28.1%), with the highest scores in the dimension of FWF and relatively low scores in the dimensions of WFC and FWC. We named this profile as the family priority group. Profile 2 comprised 605 cases (46.8%), with relatively balanced scores across the four dimensions of WFBS. We named this profile as the balanced adaptation group. Profile 3 comprised 324 cases (25.1%), with high scores in all dimensions of WFBS. We named this profile as the challenge coping group.

### 3.3. Characteristics of Three WFB Profiles

The three profiles of WFB showed significant differences in age, gender, marital status, number of children, years of work experience, education, employment type, professional title, department, personal annual income, nonclinical work stress, organizational identification, self-efficacy, and work meaning (*p* < 0.05), as shown in Table 1.

The family priority group (Profile 1) comprised a high proportion of nurses being female, aged 45 or above, having a high educational level, high seniority, high professional titles, married, with one child, employed in public institutions, having annual income between 100,000 and 200,000 yuan, engaged in internal medicine, low nonclinical work stress, and high scores in organizational identity and work meaning. The balanced adaptation group (Profile 2) showed a high proportion of nurses aged between 30 and 44 years, having worked for 6–20 years, having two children, possessing the title of senior nurse or supervisor nurse, earning a high income, facing greater nonclinical work stress, and scoring lower in organizational identification, self-efficacy, and work meaning. The challenge coping group (Profile 3) contained a high proportion of nurses being male, aged 29 or below, having a low educational level, having a short time of experience, having primary titles, low income, unmarried, contract employment, serving in departments of surgery, obstetrics and gynecology, pediatrics, and specialized departments (ICU/operating room/emergency), having low nonclinical work stress but scoring high in self-efficacy.

### 3.4. Factors Influencing the Three WFB Profiles

Using significant variables in the univariate analysis as independent variables and WFB profiles as dependent variables, an unordered multinomial logistic regression analysis was conducted. As indicated in Table 3, professional title, department, nonclinical work stress, organizational identification, work meaning, and self-efficacy score were factors influencing the latent profiles of WFB (*p* < 0.05).

In the multivariate analysis, Profile 1 and Profile 2 were compared with Profile 3, showing that a primary title (OR = 0.151, 0.27), working in surgery, pediatric, and specialized departments (ICU/operating room/emergency) (OR = 0.417, 0.367; OR = 0.365, and 0.34; OR = 0.321, 0.443), and a higher self-efficacy (OR = 0.931, 0.903) were more associated with Profile 3. Simultaneously, lower nonclinical work stress (OR = 2.769; OR = 6.926; and OR = 6.273), higher organizational identification scores (OR = 1.039), and higher sense of work meaningfulness scores (OR = 1.201) were more associated with Profile 1.

## 4. Discussion

Based on the LPA, this study identified three distinct WFB profiles of clinical nurses in China, showing notable differences in demographic characteristics, work–family characteristics, organizational identification, work meaning, and self-efficacy. These three profiles differ from those found in the previous research [9, 10], potentially due to variations in the measurement scales and characteristics of included nurses. It is recommended that managers adequately identify nurses exhibiting varying levels of WFB to ensure the interventions are both effective and specific.

In this study, the overall score for nurses' WFB was 44.35 ± 8.693, indicating a moderate level. FWF is the most significant indicator with a mean and SD of 3.94 ± 0.812, followed by WFC, WFF, and FWC, with means and SDs of 3.235 ± 1.018, 3.2 ± 0.958, and 2.495 ± 1.059, respectively. This aligns with previous studies [40, 41], reaffirming that the positive impact of family on work outweighs the negative impact of work on family and that the WFC is more prominent than FWC. In line with the spillover theory [11], this study confirms that clinical nurses' positive feelings in the family can significantly benefit their psychology at work. Additionally, the effect of negative work-related factors on nurses' family is stronger than that of negative family-related factors on work [13, 41–43]. This may be attributed to several factors. First, the family members of clinical nurses can provide support in handling family affairs, which reduces the effect of family-related factors on work. Second, the significant workload and frequent overtime demands placed on clinical nurses, coupled with additional teaching responsibilities, often render it difficult for them to balance their work and family concurrently.

We noticed that family was prioritized in Profile 1, and nurses in this profile could construct a mutually reciprocal balance between work and family. The existing literature suggests that nurses with high professional titles [44] and high education levels [45] tend to face lower WFC, owing to their rich work experience, professionalism, and positive work attitudes. This enables them to properly arrange family affairs and gain the support of their family members, thereby reducing the risk of work–family imbalance [46]. In addition, in the context of COVID-19 pandemic, more family members share the housework of nurses, thus reducing the effect of family-related factors on nurses' work [47]. A good family income allows the establishment of WFB [48, 49], which helps to alleviate work pressure, enhance self-efficacy and professional achievement, and inhibit the tendency to leave. Therefore, managers should encourage senior nurses to share their work experiences and work–family management strategies. At the same time, family–friendly policies, such as flexible scheduling, childcare services, and extended parental leave, should be provided for married nurses [50].

We found that conflicts appeared between work and family in Profile 2, but nurses could still manage to strike a balance through adaptation. Work-related factors may bring them conflicts with their family, but they can also derive energy from families to cope with. Nurses with longer working years (such as senior nurses or supervisor nurses) tend to face higher WFC due to work stress [51, 52]. This could be attributed to their increasing pressure and responsibilities in clinical work. A high salary enables nurses to support the family more [53], thereby reducing WFC. We also observed that these nurses encountered substantial nonclinical work pressure and exhibited relatively low organizational identification and self-efficacy. Consequently, nurses should communicate with their families promptly to obtain solutions and comfort when they encounter troubles at work. Hospitals or governments should take measures to relieve the effects of these factors on family, such as improving the traffic system to reduce time for commuting between hospitals and addresses [54].

We found a contradictory state between work and family in Profile 3. Young nurses recognize that WFB can alleviate stress and should be prioritized [27]. Single nurses may experience a lower risk of WFC than married nurses [12]. However, other studies have found that living with family may protect nurses' family lives [14]. This is likely due to the lack of channels for single nurses to confide and share work stress. Additionally, single nurses need to make greater compromises between work and family when responding to emergencies, which increases the risk of WFC. During the COVID-19 pandemic, young nurses undertook a majority of nursing workload and nonclinical tasks. Their lack of work experience and ability, separation from their families, and continual trainings impose them with greater emotional demands and work–life interference [21]. Nurses' higher psychological pressure and stronger intention to leave are closely related to a lower chance of WFB [19]. Hospitals and governments should take measures to enhance the satisfaction with work and self-efficacy of younger or single nurses with low educational levels and professional titles. These initiatives could include the establishment of a multilevel communication mechanism, fostering an understanding of nurses' work attitudes, promptly addressing individual needs, and thoughtfully incorporating flexible working hours [55].

Compared to Profile 3, Profile 1 contained a larger proportion of nurses with a lower nonclinical work stress and a higher organizational identity and work meaning. This finding aligns with the research by Mache et al. [56] and Yang et al. [24], suggesting that WFC positively correlates with high workload, while organizational identification and work meaningfulness reduce the risk of WFC. The spillover theory posits a bidirectional influence between family and work. Studies have found that during the COVID-19 pandemic, the medical nursing knowledge possessed by nurses can help them gain more family support, enhance their self-efficacy, relieve their work stress and burnout, and reduce their tendency to leave [57]. The alleviation of nonclinical work stress enables nurses to devote more energy and time to their family lives, promoting the balance between work and family. The research has shown that stronger organizational identification and job meaningfulness play a positive role in achieving WFB [24]. Nursing managers' pluralistic leadership styles facilitate the creation of a positive organizational atmosphere. Organizational identification can be achieved by hospitals through carrying out family activities, improving nurses' welfare, and providing more career-advancing and self-fostering opportunities [58]. In addition, nonclinical work should be reduced as much.

Nurses with a primary title in surgery, pediatric, and specialized departments (ICU/operating room/emergency) and a higher self-efficacy are more likely to belong to Profile 3. A survey of 426 clinical nurses has showed that younger nurses exhibit a lower chance of WFB than older nurses [59]. This could be due to the fact that they are at the starting stages of their careers and lives with an immature concept of WFB. Nurses in specialized departments (emergency, intensive care, and operating rooms) are susceptible to WFC than those in other departments [60]. The WFC is at a medium level, and the WFC score is significantly higher than the FWC score in nurses from these departments [25]. This may be attributed to the heavier workload and mental pressure in nursing critically ill patients. Self-efficacy positively correlates with the possibility of WFF [61]. A higher self-efficacy can enhance nurses' confidence in dealing with work and family challenges. In view of this, it is recommended that nurses in specialized departments should strengthen their self-efficacy by allocating their energy reasonably and carrying out family cycle and career development planning carefully, thereby reaching a status of WFB.

## 5. Limitations

The present study is also subjected to several limitations. First, the sample in this study is relatively limited and may not adequately represent the entire nursing population. A multicenter, multiregional, and larger sample size investigation should be performed. Second, this study is cross-sectional. Although it can reveal the association between variables, it cannot directly infer causal relationships and evolutionary processes between variables. Future longitudinal studies could provide a more comprehensive perspective on the WFC among clinical nurses. Third, self-report methods may compromise the reliability. Last, the study only focuses on the factors influencing WFC among clinical nurses, neglecting their intervention effects. Future research should design and implement intervention studies to evaluate the effects and applicability of different intervention strategies, providing scientific evidence and guidance for practical applications.

## 6. Conclusion

This study reveals that the overall level of WFB among nurses is moderate. Three profiles of WFB, including the family priority group, the balanced adaptation group, and the challenge coping group are identified in the clinical nurses, with the majority in the balanced adaptation group. The profile of WFB is affected by factors such as professional title, department, nonclinical work stress, organizational identification, work meaning, and self-efficacy. Over time, nurses' work–family status transitions to an optimal state through adjustment and coping. Hospitals and governments should take targeted measures to increase the possibility of WFB.

## 7. Implications for Nursing Management

Theoretically, employing the spillover theory as a conceptual framework and utilizing LPA, this study uncovered three latent profiles of WFB. This classification provides a novel theoretical lens for understanding the work–family relationships among nurses. Practically, we identified factors influencing the profile of WFB, which can be targeted to manage work–family relationships of nurses, particularly for young nurses working in surgery, obstetrics and gynecology, pediatrics, and specialized departments. For nursing management, tailoring strategies to group characteristics is vital. Several key measures can be implemented to alleviate the WFC faced by nurses, including fostering a good work environment, implementing flexible work schedules, and family-friendly policies, conducting regular training sessions.

## Figures and Tables

**Table 1:** Demographic characteristics of nurses and their single-factor analysis on WFB profiles.

Variables		Overall (*n* = 1292)	Family priority group (*n* = 363)	Balanced adaptation group (*n* = 605)	Challenge coping group (*n* = 324)	*F*/χ^2^	*p* value
Gender	Male	60 (4.6)	7 (1.9)	27 (4.5)	26 (8)	14.452	0.001⁣^∗^
	Female	1232 (95.4)	356 (98.1)	578 (95.5)	298 (92)		

Age (year)	≤ 29	379 (29.3)	89 (24.5)	128 (21.2)	162 (50)	93.686	< 0.001⁣^∗∗^
	30–44	518 (40.1)	144 (39.7)	274 (45.3)	100 (30.9)		
	≥ 45	395 (30.6)	130 (35.8)	203 (33.6)	62 (19.1)		

Education	Associate degree and below	242 (18.7)	42 (11.6)	110 (18.2)	90 (27.8)	30.225	< 0.001⁣^∗∗^
	Bachelor's degree	1031 (79.8)	315 (86.8)	485 (80.2)	231 (71.3)		
	Master's degree and above	19 (1.5)	6 (1.7)	10 (1.7)	3 (0.9)		

Years of work experience (year)	0–5	311 (24.1)	67 (18.5)	96 (15.9)	148 (45.7)	120.418	< 0.001⁣^∗∗^
	6–10	213 (16.5)	57 (15.7)	120 (19.8)	36 (11.1)		
	11–15	189 (14.6)	50 (13.8)	99 (16.4)	40 (12.3)		
	16–20	131 (10.1)	35 (9.6)	67 (11.1)	29 (9)		
	≥ 21	448 (34.7)	154 (42.4)	223 (36.9)	71 (21.9)		

Marital status	Married	922 (71.4)	288 (79.3)	470 (77.7)	164 (50.6)	97.985	< 0.001⁣^∗∗^
	Single	347 (26.9)	67 (18.5)	125 (20.7)	155 (47.8)		
	Others	23 (1.8)	8 (2.2)	10 (1.7)	5 (1.5)		

Number of children	0	419 (32.4)	91 (25.1)	162 (26.8)	166 (51.2)	75.992	< 0.001⁣^∗∗^
	1	725 (56.1)	238 (65.6)	360 (59.5)	127 (39.2)		
	2	148 (11.5)	34 (9.4)	83 (13.7)	31 (9.6)		

Employment type	Bianzhi nurses	540 (41.8)	173 (47.7)	265 (43.8)	102 (31.5)	29.921	< 0.001⁣^∗∗^
	Contract-based nurses	516 (39.9)	125 (34.4)	252 (41.7)	139 (42.9)		
	Others	236 (18.3)	65 (17.9)	88 (14.5)	83 (25.6)		

Professional title	Primary nurse	223 (17.3)	30 (8.3)	55 (9.1)	138 (42.6)	202.425	< 0.001⁣^∗∗^
	Senior nurse	363 (28.1)	111 (30.6)	195 (32.2)	57 (17.6)		
	Supervisor nurse	461 (35.7)	132 (36.4)	234 (38.7)	95 (29.3)		
	Cochief nurse and above	245 (18.9)	90 (24.8)	121 (20)	34 (10.5)		

Hospital level	Tertiary hospitals	1073 (83)	311 (85.7)	504 (83.3)	258 (79.6)	4.497	0.106
	Nontertiary hospitals	219 (17)	52 (14.3)	101 (16.7)	66 (20.4)		

Annual income (ten thousand yuan/year)	≤ 10	430 (33.3)	85 (23.4)	173 (28.6)	172 (53.1)	80.425	< 0.001⁣^∗∗^
	10–20	778 (60.2)	254 (70)	385 (63.6)	139 (42.9)		
	> 20	84 (6.5)	24 (6.6)	47 (7.8)	13 (4)		

Department	Internal medicine	401 (31)	122 (33.6)	198 (32.7)	81 (25)	63.520	< 0.001⁣^∗∗^
	Surgery	302 (23.4)	87 (24)	130 (21.5)	85 (26.2)		
	Obstetrics and gynecology	98 (7.6)	23 (6.3)	47 (7.8)	28 (8.6)		
	Pediatrics	70 (5.4)	14 (3.9)	24 (4)	32 (9.9)		
	Specialized departments (ICU/operating room/emergency)	211 (16.3)	40 (11)	96 (15.9)	75 (23.1)		
	Others	210 (16.3)	77 (21.2)	110 (18.2)	23 (7.1)		

Commute time (minutes)	≤ 30	785 (60.8)	231 (63.6)	359 (59.3)	195 (60.2)	10.087	0.121
	31–60	408 (31.6)	102 (28.1)	193 (31.9)	113 (34.9)		
	61–90	47 (3.6)	13 (3.6)	29 (4.8)	5 (1.5)		
	> 90	52 (4)	17 (4.7)	24 (4)	11 (3.4)		

Nonclinical work stress	Very low	90 (7)	23 (6.3)	20 (3.3)	47 (14.5)	124.917	< 0.001⁣^∗∗^
	Lower	154 (11.9)	64 (17.6)	40 (6.6)	50 (15.4)		
	Neutral	540 (41.8)	191 (52.6)	256 (42.3)	93 (28.7)		
	Higher	389 (30.1)	69 (19)	218 (36)	102 (31.5)		
	Very high	119 (9.2)	16 (4.4)	71 (11.7)	32 (9.9)		

OI (M ± SD)		19.49 ± 5.657	21.31 ± 5.404	18.54 ± 6.114	19.23 ± 4.451	28.28	< 0.001⁣^∗∗^

GSES (M ± SD)		28.05 ± 7.185	30.17 ± 6.1	25.19 ± 6.79	30.99 ± 7.03	103.638	< 0.001⁣^∗∗^

WAMI (M ± SD)		39.04 ± 6.938	43.77 ± 5.331	36.46 ± 7.151	38.56 ± 5.205	175.541	< 0.001⁣^∗∗^

Abbreviations: GSES, Generalized Self-Efficacy Scale; M, mean; OI, Organizational Identification Scale; SD, standard deviation; WAMI, the work and meaning inventory.

⁣^∗^*p* < 0.0.05.

⁣^∗∗^*p* < 0.001.

**Table 2:** Model fitting information for the latent profile of nurses' WFB.

Model	*K*	AIC	BIC	aBIC	Entropy	*p* value		Class probability (%)
						LMR	BLRT	
1	28	54,917.466	55,062.056	54,973.114	—	—	—	—
2	43	51,069.531	51,291.581	51,154.991	0.904	< 0.001⁣^∗∗^	< 0.001⁣^∗∗^	0.66/0.34
3	58	49,022.946	49,322.455	49,138.218	0.892	< 0.001⁣^∗∗^	< 0.001⁣^∗∗^	0.28/0.47/0.25
4	73	47,879.164	48,256.132	48,024.247	0.926	< 0.001⁣^∗∗^	< 0.001⁣^∗∗^	0.04/0.44/0.29/0.23
5	88	46,988.733	47,443.16	47,163.628	0.905	0.0065⁣^∗^	0.0068⁣^∗^	0.04/0.24/0.28/0.21/0.23

Abbreviations: aBIC, adjusted Bayesian information criterion; AIC, Akaike information criterion; BIC, Bayesian information criterion; BLRT, bootstrapped likelihood ratio test; LM, Lo–Mendell–Rubin.

⁣^∗^*p* < 0.0.05.

⁣^∗∗^*p* < 0.001.

**Table 3:** Multifactor analysis of three latent profiles in nurses with work–family conflict.

Variables		Family priority group (vs. challenge coping group)				Balanced adaptation group (vs. challenge coping group)			
		Odds ratio	*p* value	95% CI		Odds ratio	*p* value	95% CI	
				Lower	Upper			Lower	Upper
Gender	Male	0.697	0.482	0.255	1.904	0.902	0.78	0.438	1.859
	Female (reference)	—	—	—	—	—	—	—	—

Age (year)	≤ 29	1.703	0.43	0.455	6.378	0.439	0.163	0.138	1.396
	30–44	1.876	0.149	0.799	4.404	0.946	0.887	0.437	2.047
	≥ 45 (reference)	—	—	—	—	—	—	—	—

Education	Associate degree and below	0.499	0.399	0.099	2.507	0.881	0.865	0.204	3.801
	Bachelor's degree	0.587	0.497	0.127	2.725	0.788	0.739	0.194	3.201
	Master's degree and above (reference)	—	—	—	—	—	—	—	—

Years of work experience(year)	0–5	0.803	0.771	0.183	3.524	1.676	0.437	0.455	6.17
	6–10	0.593	0.369	0.189	1.856	1.461	0.461	0.534	4.001
	11–15	0.48	0.143	0.18	1.282	1.253	0.611	0.526	2.986
	16–20	0.521	0.171	0.205	1.325	1.056	0.898	0.458	2.435
	≥ 21 (reference)	—	—	—	—	—	—	—	—

Marital status	Married	1.435	0.585	0.393	5.245	1.406	0.573	0.43	4.601
	Single	0.753	0.731	0.149	3.794	0.796	0.76	0.185	3.433
	Others (reference)	—	—	—	—	—	—	—	—

Number of children	0	2.177	0.177	0.704	6.737	1.82	0.224	0.693	4.781
	1	1.3	0.416	0.691	2.444	0.825	0.473	0.489	1.394
	2 (reference)	—	—	—	—	—	—	—	—

Employment type	Bianzhi nurses	0.769	0.456	0.385	1.535	1.28	0.428	0.695	2.359
	Contract-based nurses	0.727	0.231	0.431	1.225	1.351	0.194	0.858	2.125
	Others (reference)	—	—	—	—	—	—	—	—

Professional title	Primary nurse	0.151	0.002⁣^∗^	0.047	0.488	0.27	0.013⁣^∗^	0.097	0.755
	Senior nurse	0.877	0.772	0.361	2.132	1.015	0.971	0.463	2.226
	Supervisor nurse	0.595	0.092	0.326	1.088	0.676	0.158	0.393	1.164
	Cochief nurse and above (reference)	—	—	—	—	—	—	—	—

Annual income (ten thousand yuan/year)	≤ 10	0.98	0.966	0.389	2.467	0.548	0.141	0.246	1.22
	10–20	1.075	0.862	0.474	2.437	0.613	0.181	0.299	1.255
	> 20 (reference)	—	—	—	—	—	—	—	—

Department	Internal medicine	0.591	0.104	0.313	1.114	0.652	0.151	0.363	1.168
	Surgery	0.417	0.008⁣^∗^	0.218	0.796	0.367	0.001⁣^∗^	0.203	0.665
	Obstetrics and gynecology	0.595	0.233	0.253	1.398	0.775	0.507	0.366	1.645
	Pediatrics	0.365	0.041⁣^∗^	0.139	0.959	0.34	0.012⁣^∗^	0.148	0.785
	Specialized departments (ICU/operating room/emergency)	0.321	0.002⁣^∗^	0.157	0.657	0.443	0.012⁣^∗^	0.235	0.835
	Others (reference)	—	—	—	—	—	—	—	—

Nonclinical work stress	Very low	2.769	0.047⁣^∗^	1.016	7.549	0.48	0.096	0.202	1.139
	Lower	6.926	< 0.001⁣^∗∗^	2.976	16.119	0.748	0.42	0.37	1.513
	Neutral	6.273	< 0.001⁣^∗∗^	3.02	13.031	1.624	0.078	0.948	2.784
	Higher	1.957	0.08	0.924	4.146	1.175	0.554	0.689	2.006
	Very high (reference)	—	—	—	—	—	—	—	—

OI		1.039	0.031⁣^∗^	1.003	1.076	1.024	0.132	0.993	1.056

GSES		0.931	< 0.001⁣^∗∗^	0.902	0.961	0.903	< 0.001⁣^∗∗^	0.879	0.928

WAMI		1.201	< 0.001⁣^∗∗^	1.156	1.247	0.983	0.226	0.956	1.011

Abbreviations: CI, confidence interval; GSES, Generalized Self-Efficacy Scale; OI, Organizational Identification Scale; WAMI, the work and meaning inventory.

⁣^∗^*p* < 0.0.05.

⁣^∗∗^*p* < 0.001.

## Data Availability

The data that support the findings of this study are available from the corresponding author upon reasonable request.
